# Parathyroid diseases and metabolic syndrome

**DOI:** 10.1007/s40618-023-02018-2

**Published:** 2023-02-11

**Authors:** R. Modica, A. Liccardi, R. Minotta, E. Benevento, G. Cannavale, A. Colao

**Affiliations:** 1 Department of Clinical Medicine and Surgery, Endocrinology Unit of Federico II University of Naples, 80131 Naples, Italy; 2 UNESCO Chair on Health Education and Sustainable Development, Federico II University of Naples, 80131 Naples, Italy

**Keywords:** Parathyroid disease, Metabolic syndrome, Hyperparathyroidism, Hypoparathyroidism, Pseudohypoparathyroidism, Obesity

## Abstract

**Purpose:**

Parathyroid diseases are related to parathyroid hormone (PTH) dysregulation by parathyroid cells or alteration of PTH function. They include hyperparathyroidism (PTH excess), hypoparathyroidism (PTH deficiency) and pseudohypoparathyroidism (PTH resistance). Little is known about correlation between parathyroid diseases and metabolic syndrome (MetS).

**Methods:**

An electronic-based search using PubMed was performed until October 2022 and articles were selected based on relevance of title, abstract, English language and publication in peer-reviewed journals.

**Results:**

Possible association between PTH alterations and the diverse manifestation of MetS have been proposed and it could be supposed that MetS may negatively influence parathyroid diseases. Available data show significant association for hyperparathyroidism and pseudohypoparathyroidism.

**Conclusions:**

This review highlights the possible implications between MetS and parathyroid diseases. Given the increasing MetS global prevalence and the higher parathyroid diseases awareness and diagnosis, it may be interesting to further explore the possible role of alterations in parathyroid homeostasis in the development of MetS components with dedicated prospective studies.

## Introduction

Parathyroid diseases and metabolic syndrome are common clinical conditions that could share common pathways, interesting to investigate. Alterations in PTH homeostasis include: hyperparathyroidism (hyperPTH), hypoparathyroidism (hypoPTH) and pseudohypoparathyroidism (pseudoPTH) Table 1 [1–10].

**Table 1:** Parathyroid diseases. *hyperPTH* hyperparathyroidism, *hypoPTH* hypoparathyroidism, *pseudoPTH* pseudohypoparathyroidism, *AHO* Albright osteodystrophy

	Pathogenesis	Biochemical features	Etiology	Prevalence
HyperPTH	Overproduction of PTH	Hypercalcemia and hypophosphatemia	Primary: parathyroid adenoma or hyperplasia Secondary: renal failure/Vitamin D deficiency Tertiary: longstanding secondary hyperPTH	86/100.000
HypoPTH	Absence or insufficient PTH production	Hypocalcemia and hyperphosphatemia	Neck surgery Autoimmune diseases Genetic disorder	37/100.000
PseudoPTH	PTH resistance	Hypocalcemia and hyperphosphatemia	Gsa gene defect Ia: AHO, multiple hormone resistance Ib: PTH resistance Ic: AHO, multiple hormone resistance Defect in cAMP production II: PTH resistance	1.1/100.000

Metabolic syndrome (MetS) is a set of diseases of high cardiometabolic risk, with steadily increasing incidence in the adult population, considerable social impact and health care costs [11]. It depends on geographic and sociodemographic factors, European MetS prevalence has been estimated as 41% in men and 38% in women [12].The most recent International Diabetes Federation (IDF) criteria states that central obesity (waist circumference ≥ 94 cm for men and ≥ 80 cm for women) allows the diagnosis of metabolic syndrome along with two of the following [13]: systolic blood pressure ≥ 130 mmHg and/or diastolic blood pressure ≥ 85 mmHg, fasting blood glucose ≥ 100 mg/dl (5.6 mmol/L), HDL cholesterol < 40 mg/dl in men or < 50 mg/dl in women, triglycerides > 150 mg/dl. Therefore, MetS is characterized by the coexistence of abdominal obesity, atherogenic dyslipidemia, elevated blood pressure and glucose alterations, which together increase the risk of developing chronic conditions such as type 2 diabetes mellitus and cardiovascular disease [14]. Parathyroid hormone (PTH) is involved in the calcium homeostasis, increasing calcium resorption from the skeleton and calcium kidney resorption. Both metabolic and cardiovascular complications of parathyroid diseases may share common pathogenetic mechanisms with the features of MetS [1, 15]. Although kidney and bone represent the primary target organs for PTH, many additional tissues also express PTH receptors, thus additional effects of PTH should be considered [1]. PTH promotes phospholipase C-b, increasing the amount of free intracellular calcium in both adipocytes and skeletal muscle [16]. There is evidence that the increase of intracellular calcium in adipocytes interferes with insulin-stimulated glucose uptake. Furthermore, in view of the common progenitor between adipocytes and osteoblasts, it has been postulated that PTH might play a role on their differentiation [16]. Noteworthy, a possible association between elevated PTH levels and increased body weight has been proposed by a meta-analysis in which body weight values were elevated in both hypercalcemic and eucalcemic populations with hyperPTH [17]. Also, Elevated PTH levels may promote hypertension, due to its correlation with aldosterone levels PTH could directly stimulate aldosterone synthesis. PTH may also contribute to endothelial dysfunction increasing peripheral vascular resistance [18]. The current review provides an overview of the available evidence regarding the possible interplay between PTH diseases and MetS or its components.

## Materials and methods

We performed an electronic-based search using PubMed until October 2022. Literature search was systematically performed through online databases including MEDLINE (via PubMed). The entree terms included “metabolic syndrome”, “parathyroid disease”, “hyperparathyroidism” “hypoparathyroidism”, “pseudoparathyroidism”, “obesity”, “diabetes mellitus”, “dyslipidemia”, “PTH”. This was complemented by a carefully handsearching reference to find additional studies and expand the search. The articles were selected based on relevance of title and abstract, English language and publication in peer-reviewed journals. Primary studies and case series dealing with patients affected by parathyroid diseases and reporting data on metabolic aspects were included.

### Hyperparathyroidism

#### Cardiovascular diseases and mortality

HyperPTH has been associated with different features of MetS. An increased prevalence of overweight, impaired glucose tolerance (IGT), dyslipidemia and hypertension has been reported both in asymptomatic and overt hyperPTH. In these patients, hypercalcemia and metabolic syndrome could play a role in cardiovascular risk. In detail, chronic hypercalcemia may cause severe and symptomatic presentations, leading to the development of hypertension, myocardial hypertrophy and shortening of QT interval with arrhythmia [19]. Recent studies (Table 2) have investigated the relationship between hyperPTH and cardiovascular mortality. In a cohort of 172 patients with hyperPTH that didn’t undergo to surgery and with hypercalcemia, cardiovascular mortality was higher compared to normocalcemic controls and was correlated with hypercalcemia with a hazard ratio (HR) of 1.72 (95% CI, 1.24–2.37; *p* < 0.001). Cardiovascular diseases were significantly over-expressed as causes of death in hypercalcemic patients, while hyperPTH patients presented an increased prevalence of features of MetS, such as hypertension, diabetes, and dyslipidemia [20]. Parathyroid surgery could improve the cardiovascular outcome of hyperPTH patients, however there are few randomized clinical trials. A recent review focused on the effect of parathyroidectomy on cardiovascular risk in hyperPTH patients, finding evidence that parathyroidectomy could be associated with a reduced cardiovascular mortality, especially in hypercalcemic patients [21]. Moreover, it has been demonstrated that patients with hyperPTH have substantial cardiac structural and functional abnormalities, such as diastolic dysfunction, valve defect and myocardial calcifications, suggesting to focus on cardiovascular follow-up with periodic echocardiography [22].

**Table 2:** Studies evaluating hyperparathyroidism and metabolic syndrome

	Study	Year	Disease	Population	Age (years)	Variables	Results
Lundgren et al. [20]	Prospective case–control study	2001	Hypercalcemic hyperparathyroidism	172 hypercalcemic patients, 344 normocalcemic controls	28–86	Serum calcium, serum PTH, CV causes of death	CV diseases were significantly over-represented as causes of death in the hypercalcemic patients
Tran et al. [23]	Cross-sectional review of records	2014	Primary hyperparathyroidism	247 patients with hyperparathyroidism (123 obese and 124 non obese)	57 ± 10	Serum PTH, obesity (BMI ≥ 30 kg/m2), nephrolithiasis and osteoporosis	Obesity is a risk factor for hypercalciuria and nephrolithiasis and is protective against osteoporosis in hyperparathyroidism patients
Yuan et al. [24]	Cross-sectional study	2021	Primary hyperparathyroidism	192 patients with hyperparathyroidism, 202 controls	55 (46–63) in hyperparathyroidism population, 49 (38–59) in controls	Serum calcium, 25OH-D, PTH, lipids profiles; bone mineral density; fat distribution	Inverted U-shape relationship between PTH and body weight and BMI
Khaleeli et al. [26]	Prospective observational study	2006	Primary hyperparathyroidism	54 patients with hyperparathyroidism	65 ± 11	Serum calcium, PTH, 75 g OGTT before and after surgery	After successful parathyroidectomy fasting and 2-h plasma glucose fall significantly; DM and IGT/IFG often ameliorates to IGT or NGT
Kumar et al. [28]	Cross-sectional study	1993	Primary hyperparathyroidism	19 patients with hyperparathyroidism, 11 age and BMI matched controls	54 (41–59) in hyperparathyroidism population, 54 (42–61) in controls	Serum calcium, PTH, plasma glucose and C-peptide before and after glucose infusion	Insulin insensitivity is present in hyperparathyroidism and may be the cause of glucose intolerance and diabetes
Procopio et al	Observational case–control study	2002	Primary hyperparathyroidism	59 patients with hyperparathyroidism and no DM, 60 controls	59 (55.3–62.2) in hyperparathyroidism population, 57 (50.8–60.1) in controls	Serum calcium, PTH, 75 g OGTT	Increased insulin resistance and prevalence of IGT and undiagnosed diabetes in hyperparathyroidism patients
Ejlsmark-Svensson et al. [29]	Randomized clinical trial	2019	Primary hyperparathyroidism	79 patients with hyperparathyroidism	64 (56–69)	24-h BP and fasting plasma cholesterol levels at baseline and 3 months after surgery	PTX may decrease risk of CV diseases in hyperparathyroidism by lowering total cholesterol levels; ambulatory diastolic BP increases in response to surgery
Norenstedt et al. [30]	Randomized double-blind clinical trial	2013	Primary hyperparathyroidism	150 patients with hyperparathyroidism	60 (30–80)	Metabolic profile, blood pressure and 25OH-D at baseline and 12 months after surgery	PTX proved effective in reducing insulin resistance
Hagström et al. [31]	Observational case–control study	2001	Primary hyperparathyroidism	87 patients with hyperparathyroidism, 87 controls	66.7 ± 5.74 in hyperparathyroidism population, 66.9 ± 5.66 in controls	Serum lipids, lipoprotein fractions and influences of treatment for the parathyroid disease	Proatherosclerotic dyslipidemia characterizes mild hyperparathyroidism and is effectively reversed by PTX
Heyliger et al. [33]	Retrospective observational study	2009	Primary hyperparathyroidism	368 patients with hyperparathyroidism	52 ± 13	Serum calcium, PTH, BP	PTX in hypertensive patients reduces both systolic and diastolic BP
Broulik et al. [34]	Retrospective observational study	2011	Primary hyperparathyroidism	1020 patients with hyperparathyroidism, 1020 controls	58 ± 14 in hyperparathyroidism population, 60 ± 15 in controls	BP	PTX in hypertensive patients reduce systolic and diastolic BP
Graff-Baker et al. [35]	Cohort study	2019	Primary hyperparathyroidism	2380 patients with hyperparathyroidism, 501 with PTX and 1879 with no surgery	65.3 ± 9.7 in PTX population, 71.9 ± 10.4 in no surgery population	BP and antihypertensive medications use	PTX is associated with decreases in BP and with reduced requirements for antihypertensive medications
Parfrey et al. [37]	Global, multicenter, randomized placebo-controlled trial	2015	Primary hyperparathyroidism	3883 patients in hemodialysis and treatment with cinacalcet in two arms (< and ≥ 65 years)	50 (32–61) in < 65 years arm, 71 (66–80) in ≥ 65 years arm	Death, major CV events	Cinacalcet decreased the risk of death and of major CV events in older, but not younger, patients with moderate to severe hyperparathyroidism receiving hemodialysis
Purra et al. [22]	Prospective case–control study	2021	Primary hyperparathyroidism	100 patients with primary hyperparathyroidism and 113 controls	48 ± 14 in hyperparathyroidism population, 50 ± 14 in controls	Echocardiographic parameters	Symptomatic patients with hyperparathyroidism have substantial cardiac structural and functional abnormalities
Forman et al. [44]	Cross-sectional study	2010	25OH-D deficit	184 normotensive individuals	42.2 ± 9.5 in ≥ 30 ng/ml of 25OH-D; 40.0 ± 12.2 in 30–15 ng/ml of 25OH-D; 38.2 ± 13.5 in < 15 ng/ml of 25OH-D	Plasma renin activity and angiotensin II and the renal plasma flow response to infused angiotensin II; 25OH-D	Low plasma 25OH-D levels may result in upregulation of the RAS in otherwise healthy humans
Vaidya et al. [45]	Observational study	2011	25OH-D deficit and obesity	97 patients with hypertension	46.8 ± 1.2 in non obese arm, 46.1 ± 1.5 in obese arm	Plasma renin activity, 25OH-D, BP	Vascular RAS activity may progressively increase when 25OH-D deficiency occurs in obesity
McMullan et al	Randomized, double-blind, placebo-controlled trial	2017	25OH-D deficit	93 patients	39.3 ± 12.3 in vitamin D arm, 34.7 ± 11.3 in placebo arm	25OH-D, BP, RAS	No benefit from correcting vitamin D deficiency on RAS activity or BP after 8 weeks
El Hilali et al. [46]	Population-based cohort study	2015	Secondary hyperparathyroidism	1317 patients	75 (70–81)	25OH-D, PTH, CV mortality	Low serum 25OH-D is associated with overall mortality in older persons. High serum PTH is associated with a higher risk of overall mortality and CV mortality in older men

*PHT* parathormone, *25OH-D* 25-hydroxyvitamin D, *BMI* body mass index, *BP* blood pressure, *DM* diabetes mellitus, *IGT* impaired glucose tolerance, *IFG* impaired fasting glucose, *NGT* normal glucose tolerance, *PTX* parathyroidectomy, *CV* cardiovascular, *RAS* Renin-Angiotensin system

### Obesity

**Fig. 1:**
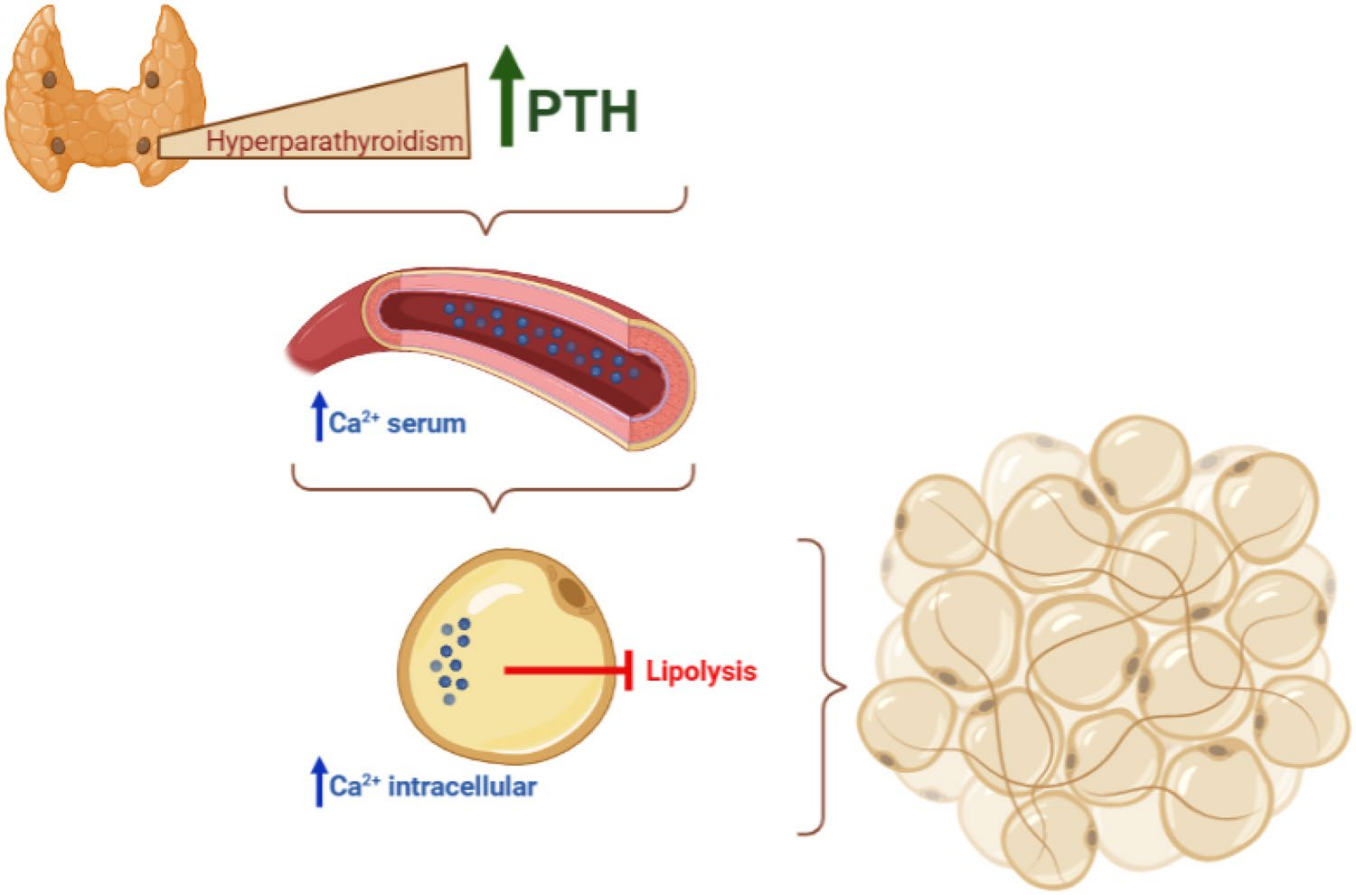
A possible mechanism that correlates hyperparathyroidism and obesity. Hypercalcemia PTH-induced may inhibit the lipolysis leading to an increase in body weight

The role of hyperPTH in obesity is highly debated. There are some evidences that hyperPTH patients present a higher prevalence of obesity compared to healthy controls. A metanalysis conducted by Bollad et al. reviewed 17 studies, analyzing data of 617 patients with primary hyperPTH and 1248 controls. HyperPTH patients were 3.34 kg heavier than controls (95% CI, 1.97–4.71; *p* < 0.00001). Further analysis demonstrated that average weight and body mass index (BMI) were higher in hyperPTH patients than controls (respectively 3.1 kg of weight and 1.1 kg/m^2^ of BMI; *p* < 0.00001) [17]. Excess of PTH can promote an increase in body weight according to a proposed mechanism in which an increased intracellular calcium concentration, induced by PTH, inhibits the lipolytic response to catecholamines in adipocytes (Fig. 1) [16]. Reversely, obesity impacts the course of hyperPTH. It has been demonstrated that obese hyperPTH patients, compared to non-obese patients, presented a higher prevalence of hypercalciuria (41 vs 23%, *p* = 0.01) and nephrolithiasis (36 vs 21%, *p* = 003), but a lower rate of osteoporosis [23]. In a recent study, authors proved a positive correlation between PTH levels and body weight, BMI and visceral adipose tissue area (VAA). Interestingly, this correlation turned to be negative in the PTH range of 147–2511 pg/ml (third tertile of their study), giving to the correlation an inverted U-shape relationship and suggesting that a marked increase in PTH levels is the cause of the decrease in body weight and BMI in these patients. This behavior could be explained as a consequence of hypercalcemia or malnutrition due to gastro-intestinal manifestation of hyperPTH. Alternatively, animal studies provided evidence that exposure to high levels of PTH can induce increased expression of thermogenesis genes, resulting in white adipose browning and muscle wasting [24, 25].

### Impaired glucose tolerance and diabetes

Prevalence of impaired glucose tolerance and diabetes in hyperPTH patients resulted higher compared with general population, with a prevalence of 25 and 10%, respectively. In particular, prevalence of impaired fasting glucose, impaired glucose tolerance and diabetes is increased in all hyperPTH patients, both symptomatic and asymptomatic [26, 27]. However, the lack of long-term prospective trials leads to a reduced understanding of glucose metabolism alterations in these patients. Several studies demonstrated that patients with hyperPTH present a reduced insulin sensitivity, basal and after stimulus [27]. The impact of therapy on glucose metabolism in these patients remains unclear, since the regression of diabetes and of impaired glucose tolerance after parathyroidectomy has been observed only in a study on 34 patients [26, 28].

### Lipid alterations

Regarding lipid metabolism, several studies have identified an atherogenic lipid profile in patients with hyperPTH, characterized by an increase in LDL cholesterol and triglycerides levels and a reduction in HDL cholesterol. However, an improvement in these parameters has not been observed after 3 [29], 6 and 12 months [30] after parathyroidectomy, even after the exclusion of patients already treated with lowering cholesterol drugs [29]. These data suggest that surgery seems to play a marginal role in dyslipidemia in hyperPTH patients. Contrasting results have been observed in patients with mild hyperPTH, since several studies find a reduction in total cholesterol, LDL cholesterol (even in patients on treatment) and triglycerides levels and an increase in HDL cholesterol levels [31]. Although parathyroidectomy improves dyslipidemia only in patients with mild hyperPTH, a role of PTH in lipid metabolism should be considered [31].

### Hypertension

In several studies, hyperPTH is associated with an increased risk of hypertension, since this feature has been observed with a prevalence of 40–65% in hyperPTH patients, higher than general population [18, 32]. Several mechanisms have been proposed to explain this relationship. Calcium is a regulator of the renin–angiotensin–aldosterone system (RAAS). Indeed, chronic hypercalcemia causes an increase in renin activity, through stimulation of Calcium Sensing Receptor (CaSR). Also, elevated PTH levels contribute to hypertension, because this hormone correlates with aldosterone levels in these patients and the possible underlying mechanism is that PTH may directly stimulate aldosterone synthesis. Moreover, PTH action on hypertension could be explained through endothelial dysfunction, increasing sympathetic activity or with a direct effect on vascular smooth muscle cells, leading to an increased peripheral vascular resistance with hypertension [18]. Several studies evaluated reversibility of cardiovascular disease and hypertension in patients with hyperPTH undergoing surgery. Indeed, patients presented a reduction of plasmatic renin activity, angiotensin and aldosterone levels, and consequently a significative reduction in systolic and diastolic blood pressure after parathyroidectomy [18]. Parathyroidectomy improved blood pressure in several studies; blood pressure improvement has been observed already 6 months after surgery in two series of 147 [33] and 726 patients [34]. This improvement is maintained over time in a study that evaluated 501 patients after 2 years from surgery [35].

### Chronic kidney diseases

The features of MetS are well-known risk factors for chronic kidney disease (CKD), while their correlation with secondary hyperPTH is still unclear [36]. Bone disease secondary to CKD is associated with an increased mortality rate and incidence of cardiovascular disease. Treatment of risk factors is not sufficient, alone, to prevent vascular calcification and to reduce cardiovascular mortality. However, as demonstrated in the EVOLVE trial for patients in dialysis, cinacalcet treatment leads to a reduction of PTH level, with no modifications of cardiovascular parameters, except for a mild reduction of blood pressure. In conclusion, hyperPTH secondary to CKD is a major determinant for the development of vascular calcifications and for the increased cardiovascular mortality [37].

### Vitamin D deficiency

Vitamin D deficiency has been proposed as a possible factor that can contribute to the development of MetS. Indeed, adipocyte, pancreatic beta cells and muscle cells are target tissues for this vitamin. Several epidemiological studies suggest an association between obesity, insulin resistance, diabetes and a reduced vitamin D activity [38]. In particular, Meng et al. observed lower total 25(OH)D and vitamin D binding protein levels (*p* < 0.001) in patients with hyperPTH [39]. Moreover, vitamin D concentrations present an inverted correlation with obesity-related parameters as BMI and waist circumference and vitamin D may contribute to build the sarcopenic obesity phenotype[40, 41]. In a similar way, vitamin D deficiency is associated with insulin resistance and diabetes, although vitamin D integration is not recommended to prevent or improve diabetes. Vitamin D deficiency has been also associated with hypertension. Several studies demonstrated that vitamin D levers are inversely correlated to renin and angiotensin II levels [42–44]. Based on these results, a trial aimed to verify the effect of correcting vitamin D deficiency, but no benefit was found on blood pressure after 8 weeks of therapy, demonstrating that vitamin D isn’t a modifiable target for lowering blood pressure in deficient patients [45]. In the evaluation of the vitamin D impact on blood pressure, some author observed that blood pressure modifications were mostly correlated with PTH levels and not with vitamin D, suggesting that hypertension is primarily influenced by PTH levels [46].

## Hypoparathyroidism and pseudohypoparathyroidism

Literature data analyzing the metabolic aspects and associated cardiovascular risk in patients with hypoPTH are lacking and mainly regard the influence of PTH treatment in these patients. On the basis of animal and human studies in which osteocalcin (OC) and undercarboxylated osteocalcin (ucOC) has been shown to regulate glucose metabolism Harsløf et al. conducted a randomized trial enrolling 62 patients with hypoPTH and assigned them double-blind to treatment with 100 g PTH (1–84) administered once daily subcutaneously or placebo for 24 weeks (24w), both groups received optimal calcium and vitamin D supplement (Table 3). Patients underwent at baseline and after 24w measures of body composition (body weight, truncal fat, and total body fat) and dosage of glucose, insulin, adiponectin, leptin, HOMA-IR, OC, and ucOC. In response to treatment, ucOC increased (*p* < 10^–50^) and body weight decreased significantly in the PTH-treated group; additionally, in the placebo group body weight increased (*p*:0.04). Changes in ucOC were inversely linked with changes in total body fat mass (*p* = 0.03) and body weight (*p* = 0.004). Therefore, PTH treatment seems to affect body weight independently of glycoinsulinemic metabolism, despite the involvement of OC/ucOC [47]. A further study analyzed the same population by investigating cardiovascular risk in terms of ECG and blood pressure (BP), showing no significant effects on BP or heart rate after 6‐month treatment. However patients allocated to rhyperPTHH(1‐84) treatment had a significantly higher heart rate at all three timepoints of measurement on the day of the 24 h study. Because this study didn’t show significant correlations between plasma magnesium/Ca_2_ levels and heart rate, it appears that the elevated heart rate is probably caused by PTH directly [48].

**Table 3:** Studies evaluating hypoparathyroidism/pseudohypoparathyroidism and metabolic syndrome

	Study	Year	Disease	Gene	Population	Age	Variables	Results	Notes
Harsløf T et al. [47]	Randomized, double-blinded, placebo-controlled prospective study	2015	Hypoparathyroidism		Cases: 28 Controls: 30	31–78	ucOC, glucose, adiponectin, leptin, homeostasis model of assessment for insulin resistance, total body fat mass, truncal fat, weight in response to 100mcg PTH (1–84) or placebo	ucOC increased (*p* < 10–50) and body weight decreased significantly in the PTH-treated group; in the placebo group body weight increased (*p*:0.04)	6‐month treatment
Sikjaer T [48]	Randomized, double-blinded, placebo-controlled prospective study	2015	Hypoparathyroidism		Cases: 28 Controls: 30	31–78	Cardiovascular risk	no significant effects on BP or heart rate	6‐month treatment
Wang et al. [51]	Cross-sectional, case–control study	2014	PHP-1A	GNAS	Cases: 10 Controls: 9 Cases: 10 Obese controls: 30 Normal weight siblings: 6	2–12	HQ CEBQ	not a significant difference Total HQ score (*p* = 0.72) and CEBQ Total HQ score (*p* = 0.40)	Caregivers reported an increased interest in food before age 2 years in 6 of 10 PHP-1a patients (60%), 9 of 30 obese controls (30%) and none of the siblings (*p* = 0.04)
Long et al. [52]	Observational study	2007	PHP-1A	GNAS	Cases: 40 Controls: 13	2–82	Obesity (BMI)	Adult sub-group with PHP1a was characterized by a 3.5-fold higher frequency of severe obesity and a twofold higher prevalence of obesity compared to the general population, additionally children sub-group with PHP1a had an approximately 5.2-fold higher frequency of obesity	
Roizen et al. [55]		2016	PHP-1A	GNAS	Cases: 12 Controls: 156	5–46	REE	REE was significantly different between participants with PHP1A and obese controls (PHP1A 1080 ± 278 kcal/d vs obese 1544 ± 357 kcal/d (mean ± sd): two-tailed t > 0.001)	
Muniyappa et al. [58]	Cross-sectional, case–control study		PHP-1A	GNAS	Cases: 8 Controls: 24	41 ± 2	Insulin sensitivity, acute insulin response to glucose, and disposition index	Nondiabetic PHP1a patients tended to have a lower SI (*p* = 0.09) and reduced OGIS (*p* = 0.03). Disposition index, a composite measure of B-cell function, also tended to be lower in patients (*p* = 0.07)	
Nwosu et al. [56]	Case report	2009	PHP-1A	GNAS		5	Insulin sensitivity	Hypocalcemia, hypothyroidism, growth-hormone deficiency and insulin resistance	skin examination revealed subcutaneous nodules and acanthosis nigricans

*PHP-1A* pseudohypoparathyroidism type 1a, *HQ* Hyperphagia Questionnaire, *CEBQ* Children's Eating Behavior Questionnaire, *REE* resting energy expenditure

**Fig. 2:**
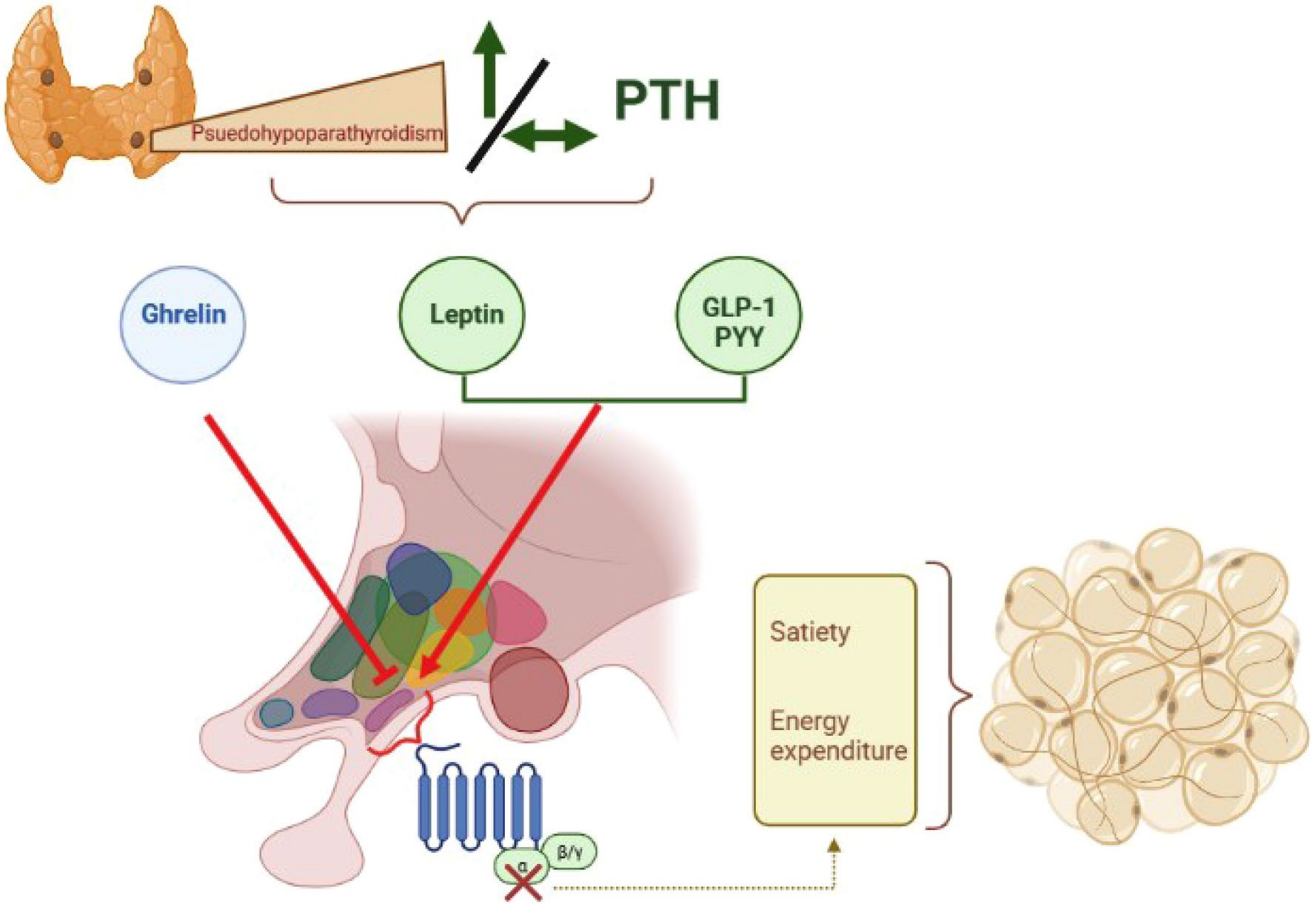
A possible mechanism that correlates PseudoPTH and obesity: Ghrelin (orexigen hormone) and Peptide YY-PYY/glucagon-like peptide 1-GLP-1 (anorexigenic hormones) act at the hypothalamic arcuate nucleus to regulate hunger/satiety signaling. Gsa and cAMP production are necessary to allow this mechanism, so their defects could bring to a satiety and energy expenditure alteration causing adipose tissue accumulation

Furthermore, valve heart calcifications are significantly associated with hyperphosphatemia in a cohort of 37 patients with hypoPTH, in particular Polonine et al. identified a possible cutoff of serum phosphorus > 5.05 mg/dl (*p*:0.05). Despite, the paucity of evidence in the literature in the area of hypoPTH, several studies have focused on metabolic aspect of pseudoPTH considering that it is often associated with obesity [49–52]. Obesity and alterations in glucose metabolism have been observed in pseudoPTH population, but a solid association has not yet been recognized. In an observational study, 53 patients with Albright hereditary osteodystrophy (AHO) were evaluated according to anthropometric data and biochemical analysis [52]. This cohort included 40 patients with pseudoPTH type 1A (PHP1a, disruption of maternal Gas allele) and 13 subjects with pseudoPTH (disruption of the paternal allele) confirmed GNAS mutated, between 2 and 82 years old (children sub-group: patients with < 18 years); almost the total PHP1a population was in optimal hormone replacement therapy, no data were available in 4 patients, and the anthropometric measurements preceded GH deficiency diagnosis. In this case, data showed that adult sub-group with PHP1a was characterized by a 3.5-fold higher frequency of severe obesity and a twofold higher prevalence of obesity compared to the general population, additionally children sub-group with PHP1a had an approximately 5.2-fold higher frequency of obesity [52]. The entire PHP1a population had a BMI z-scores of 2.31 ± 0.18 vs 0.65 ± 0.31 of pseudoPTH population (*P* 0.000032) [52]. Indeed, the prevalence of obesity was 66.7% among PHP1a adults (16.7% were severe obese, BMI > 40 kg/m^2^), while it was about the same of the general population in pseudoPTH patients and there was no severe obese. In the children sub-group, obese prevalence was 89.3% among PHP1a and no children with pseudoPTH was obese [52]. The weight gain would appear to be related to Gsa subunit activity, but it should be emphasized that obesity, together with hyperphagia, is an early clinical manifestation of pseudoPTH, so metabolic complications could be secondary to this manifestation. For this reason, a study investigated eating behavior in a cohort of 10 patients (2–10 years old) affected by PHP-1a and matched on age, gender and BMI z-score with non-sick siblings (*n*: 6) and a control group of obese (n: 30) [51]. Current use of appetite-suppressing medications and obesity genetic syndrome were exclusion criteria [51]. The three cohorts were submitted to Hyperphagia Questionnaire (HQ) [53] and Children’s Eating Behavior Questionnaire (CEBQ) [54] completed by the primary caregiver [51]. There were no differences between the PHP-1a and obese control groups; both were significantly more fat than the sibling group [51]. These results indicated that children with PHP-1a, with adequate treatment of thyroid hormone and growth hormone replacement, may not be hyperphagic when compared to other obese children because there was no statistically difference between the PHP-1a group and matched controls for total HQ score [51]. These data do not support the hypothesis that obesity in pseudoPTH could be associated with melanocortin-4 receptor (MCR4) malfunction, a Gsa signaling pathways, which can induce hyperphagia Fig. 2. [49, 55]. A further hypothesis of correlation between obesity and pseudoPTH concerns energy metabolism. Roizen et al. recruited 12 participants (between 5 and 46 years old) with a diagnosis of PHP1A and AHO features evaluating the REE (resting energy expenditure), biochemical, endocrine, and auxological parameters; controls were a cohort of 156 obese participants. Patients with PHP1A had greater decreases in REE from expected than obese controls, providing evidence of a unique and significant defect that likely accounts for early-onset obesity in this disorder [55]. Finally, among those participants that provided dietary data, they consumed significantly fewer calories than their recommended daily allowance [55]. Hence, these results provide evidence that increased BMI in PHP1A is due principally to decreased REE. Nor were any consistent effects observed between percent expected REE and age, calcium, thyroid hormone, IGF-1 standard deviation score, or GH status in those with PHP1A [55].

Regarding metabolic complications of obesity, patients with PHP1a have not frequently been reported to have insulin resistance, despite some case series [56]. Moreover, Germain-Lee et al. described a case of acanthosis nigricans in a cohort of 13 PHP1a patients with normal Hba1c and fasting insulin levels. Considering that acanthosis nigricans is not a typical feature of this condition, these may probably represent obesity related complications in PHP1a patients [57]. In a cross-sectional, case–control study, people with PHP1a were more likely to develop diabetes than those of the same age matched according to percentage of body fat [58]. Indeed, in 8 PHP1a cases hemoglobin A1c (HbA1c) and fasting plasma glucose were significantly higher than in 24 healthy controls (P: 0.04 and P:0.02, respectively) [58]. When compared to similarly obese controls, PHP1a patients had lower insulin sensitivity, indicating that factors other than obesity contribute to lower insulin sensitivity in these patients [58]. A possible mechanism involved is the participation of Gsa in the response to glucagon-like peptide 1 (GLP1) and other incretin hormones in pancreatic islet cells Fig. 2 [59].

Hence, the relationship between metabolic alterations and hypo/pseudoPTH has never been evaluated considering altered PTH homeostasis.

## Conclusions

This review highlights the possible implications between MetS and parathyroid diseases, identifying studies showing significant associations only in patient with hyperPTH or pseudoPTH. Regarding hyperPTH, all MetS’ components show significant associations, demonstrating how overweight, obesity, hypertension, diabetes and dyslipidemia are more prevalent in hyperPTH patients than in general population. However, a possible pathogenetic mechanism has been hypothesized only for obesity, as PTH-induced hypercalcemia could be responsible for the decrease in adipocyte lipolysis. Moreover, it is necessary to further clarify the role of parathyroidectomy in the improvement or resolution of MetS’ components. In pseudoPTH, the association with obesity seems to be caused by Gsa subunit alteration regulating transcriptional cascades of genes involved in the etiopathogenesis of weight gai. On the other hand, literature data are currently insufficient to clarify the role of MetS in hypoparathyroid patient.

Given the increasing MetS global prevalence and the higher parathyroid diseases awareness and diagnosis, it may be interesting to further explore the possible role of alterations in parathyroid homeostasis in the development of MetS components with dedicated prospective studies.

## Data Availability

Data sharing not applicable to this article as no data sets were generated or analyzed during the current study.

## References

[1] Bilezikian JP, Bandeira L, Khan A (2018). Hyperparathyroidism. Lancet.

[2] Clarke BL, Brown EM, Collins MT (2016). Epidemiology and diagnosis of hypoparathyroidism. J Clin Endocrinol Metab.

[3] Bilezikian JP, Khan A, Potts JT (2011). Hypoparathyroidism in the adult: epidemiology, diagnosis, pathophysiology, target-organ involvement, treatment, and challenges for future research. J Bone Miner Res.

[4] Shoback D (2008). Clinical practice. Hypoparathyroidism N Engl J Med.

[5] Marcucci G, Beccuti G, Carosi G (2022). Multicenter retro-prospective observational study on chronic hypoparathyroidism and rhPTH (1–84) treatment. J Endocrinol Invest.

[6] Marcucci G, Cianferotti L, Parri S (2018). HypoparaNet: a database of chronic hypoparathyroidism based on expert medical-surgical centers in Italy. Calcif Tissue Int.

[7] Underbjerg L, Sikjaer T, Mosekilde L (2016). Pseudohypoparathyroidism - epidemiology, mortality and risk of complications. Clin Endocrinol (Oxf).

[8] Jamal SA, Miller PD (2013). Secondary and tertiary hyperparathyroidism. J Clin Densitom.

[9] Thakker RV (2016). Genetics of parathyroid tumours. J Intern Med.

[10] Bastepe M, Jüppner H (2005). GNAS locus and pseudohypoparathyroidism. Horm Res.

[11] Saklayen MG (2018). The global epidemic of the metabolic syndrome. Curr Hypertens Rep.

[12] Gao W, DECODE Study Group (2008). Does the constellation of risk factors with and without abdominal adiposity associate with different cardiovascular mortality risk?. Int J Obes (Lond).

[13] Alberti KGMM, Zimmet P, Shaw J (2005). The metabolic syndrome–a new worldwide definition. Lancet.

[14] Bovolini A, Garcia J, Andrade MA (2021). Metabolic syndrome pathophysiology and predisposing factors. Int J Sports Med.

[15] Saha S, Mannar V, Kandasamy D (2022). Vertebral fractures, trabecular bone score and their determinants in chronic hypoparathyroidism. J Endocrinol Invest.

[16] McCarty MF, Thomas CA (2003). PTH excess may promote weight gain by impeding catecholamine-induced lipolysis-implications for the impact of calcium, vitamin D, and alcohol on body weight. Med Hypotheses.

[17] Bolland MJ, Grey AB, Gamble GD (2005). Association between primary hyperparathyroidism and increased body weight: a meta-analysis. J Clin Endocrinol Metab.

[18] Tomaschitz A, Ritz E, Pieske B (2012). Aldosterone and parathyroid hormone: a precarious couple for cardiovascular disease. Cardiovasc Res.

[19] Cormier C, Koumakis E (2022). Bone and primary hyperparathyroidism. Joint Bone Spine.

[20] Lundgren E, Lind L, Palmér M (2001). Increased cardiovascular mortality and normalized serum calcium in patients with mild hypercalcemia followed up for 25 years. Surgery.

[21] Frey S, Mirallié É, Cariou B (2021). Impact of parathyroidectomy on cardiovascular risk in primary hyperparathyroidism: a narrative review. Nutr Metab Cardiovasc Dis.

[22] Purra S, Lone AA, Bhat MH (2022). Cardiac structural and functional abnormalities in primary hyperparathyroidism. J Endocrinol Invest.

[23] Tran H, Grange JS, Adams-Huet B (2014). The impact of obesity on the presentation of primary hyperparathyroidism. J Clin Endocrinol Metab.

[24] Yuan T-J, Chen L-P, Pan Y-L (2021). An inverted U-shaped relationship between parathyroid hormone and body weight, body mass index, body fat. Endocrine.

[25] Kir S, White JP, Kleiner S (2014). Tumour-derived PTH-related protein triggers adipose tissue browning and cancer cachexia. Nature.

[26] Khaleeli AA, Johnson JN, Taylor WH (2007). Prevalence of glucose intolerance in primary hyperparathyroidism and the benefit of parathyroidectomy. Diabetes Metab Res Rev.

[27] Kumar S, Olukoga AO, Gordon C (1994). Impaired glucose tolerance and insulin insensitivity in primary hyperparathyroidism. Clin Endocrinol (Oxf).

[28] Procopio M, Magro G, Cesario F (2002). The oral glucose tolerance test reveals a high frequency of both impaired glucose tolerance and undiagnosed Type 2 diabetes mellitus in primary hyperparathyroidism. Diabet Med.

[29] Ejlsmark-Svensson H, Rolighed L, Rejnmark L (2019). Effect of parathyroidectomy on cardiovascular risk factors in primary hyperparathyroidism: a randomized clinical trial. J Clin Endocrinol Metab.

[30] Norenstedt S, Pernow Y, Brismar K (2013). Primary hyperparathyroidism and metabolic risk factors, impact of parathyroidectomy and vitamin D supplementation, and results of a randomized double-blind study. Eur J Endocrinol.

[31] Hagström E, Lundgren E, Lithell H (2002). Normalized dyslipidaemia after parathyroidectomy in mild primary hyperparathyroidism: population-based study over five years. Clin Endocrinol (Oxf).

[32] Kalla A, Krishnamoorthy P, Gopalakrishnan A (2017). Primary hyperparathyroidism predicts hypertension: results from the national inpatient sample. Int J Cardiol.

[33] Heyliger A, Tangpricha V, Weber C (2009). Parathyroidectomy decreases systolic and diastolic blood pressure in hypertensive patients with primary hyperparathyroidism. Surgery.

[34] Broulik PD, Brouliková A, Adámek S (2011). Improvement of hypertension after parathyroidectomy of patients suffering from primary hyperparathyroidism. Int J Endocrinol.

[35] Graff-Baker AN, Bridges LT, Chen Q (2020). Parathyroidectomy for patients with primary hyperparathyroidism and associations with hypertension. JAMA Surg.

[36] Zhang X, Lerman LO (2017). The metabolic syndrome and chronic kidney disease. Transl Res.

[37] Parfrey PS, Drüeke TB, Block GA (2015). the effects of cinacalcet in older and younger patients on hemodialysis: the evaluation of cinacalcet HCl therapy to lower cardiovascular events (EVOLVE) trial. Clin J Am Soc Nephrol.

[38] Barrea L, Frias-Toral E, Pugliese G (2021). Vitamin D in obesity and obesity-related diseases: an overview. Minerva endocrinology.

[39] Meng L, Su C, Shapses SA (2022). Total and free vitamin D metabolites in patients with primary hyperparathyroidism. J Endocrinol Invest.

[40] Kamwa V, Welch C, Hassan-Smith ZK (2021). The endocrinology of sarcopenia and frailty. Minerva endocrinology.

[41] Pereira-Santos M, Costa PRF, Assis AMO (2015). Obesity and vitamin D deficiency: a systematic review and meta-analysis. Obes Rev.

[42] Vaidya A, Forman JP, Hopkins PN (2011). 25-Hydroxyvitamin D is associated with plasma renin activity and the pressor response to dietary sodium intake in Caucasians. J Renin Angiotensin Aldosterone Syst.

[43] Vaidya A, Forman JP, Williams JS (2011). Vitamin D and the vascular sensitivity to angiotensin II in obese Caucasians with hypertension. J Hum Hypertens.

[44] Forman JP, Williams JS, Fisher NDL (2010). Plasma 25-hydroxyvitamin D and regulation of the renin-angiotensin system in humans. Hypertension.

[45] McMullan CJ, Borgi L, Curhan GC (2017). The effect of vitamin D on renin-angiotensin system activation and blood pressure: a randomized control trial. J Hypertens.

[46] el Hilali J, de Koning EJ, van Ballegooijen AJ (2016). Vitamin D, PTH and the risk of overall and disease-specific mortality: results of the longitudinal aging study Amsterdam. J Steroid Biochem Mol Biol.

[47] Harsløf T, Sikjær T, Sørensen L (2015). The effect of treatment with PTH on undercarboxylated osteocalcin and energy metabolism in hypoparathyroidism. J Clin Endocrinol Metab.

[48] Sikjaer T, Amstrup AK, Rolighed L (2013). PTH(1–84) replacement therapy in hypoparathyroidism: a randomized controlled trial on pharmacokinetic and dynamic effects after 6 months of treatment. J Bone Miner Res.

[49] Valette M, Bellisle F, Carette C (2013). Eating behaviour in obese patients with melanocortin-4 receptor mutations: a literature review. Int J Obes (Lond).

[50] Farooqi IS, Keogh JM, Yeo GSH (2003). Clinical spectrum of obesity and mutations in the melanocortin 4 receptor gene. N Engl J Med.

[51] Wang L, Shoemaker AH (2014). Eating behaviors in obese children with pseudohypoparathyroidism type 1a: a cross-sectional study. Int J Pediatr Endocrinol.

[52] Long DN, McGuire S, Levine MA (2007). Body mass index differences in pseudohypoparathyroidism type 1a versus pseudopseudohypoparathyroidism may implicate paternal imprinting of Galpha(s) in the development of human obesity. J Clin Endocrinol Metab.

[53] Dykens EM, Maxwell MA, Pantino E (2007). Assessment of hyperphagia in Prader-Willi syndrome. Obesity (Silver Spring).

[54] Wardle J, Guthrie CA, Sanderson S (2001). Development of the children’s eating behaviour questionnaire. J Child Psychol Psychiatry.

[55] Roizen JD, Danzig J, Groleau V (2016). Resting energy expenditure is decreased in pseudohypoparathyroidism Type 1A. J Clin Endocrinol Metab.

[56] Nwosu BU, Lee MM (2009). Pseudohypoparathyroidism type 1a and insulin resistance in a child. Nat Rev Endocrinol.

[57] Germain-Lee EL, Groman J, Crane JL (2003). Growth hormone deficiency in pseudohypoparathyroidism type 1a: another manifestation of multihormone resistance. J Clin Endocrinol Metab.

[58] Muniyappa R, Warren MA, Zhao X (2013). Reduced insulin sensitivity in adults with pseudohypoparathyroidism type 1a. J Clin Endocrinol Metab.

[59] Xie T, Chen M, Weinstein LS (2010). Pancreas-specific Gsalpha deficiency has divergent effects on pancreatic alpha- and beta-cell proliferation. J Endocrinol.

